# Epidemiology and Clinical Outcome of Patients Hospitalized With Pelvic Inflammatory Disease Complicated by Tubo-Ovarian Abscess

**DOI:** 10.1155/S1064744995000470

**Published:** 1995

**Authors:** Ying Chan, Winsome Parchment, Joan H. Skurnick, Laura Goldsmith, Joseph J. Apuzzio

**Affiliations:** 1 Department of Obstetrics and Gynecology New Jersey Medical School 185 South Orange Avenue Newark NJ 07103 USA; 2 Department of Public Health and Preventive Medicine New Jersey Medical School Newark NJ USA

## Abstract

*Objective:* The purpose of this retrospective study was to compare the clinical outcome and characteristics of pelvic inflammatory disease (PID) complicated by tubo-ovarian abscess (TOA) with PID without TOA.

*Methods:* Chart reviews were performed for all PID admissions to the University of Medicine and Dentistry of New Jersey-University Hospital, Newark, NJ, from January 1, 1992, to December 31, 1993.

*Results:* The incidence in this study of TOA based on sonographic evidence of a complex adnexal mass was 18%. The major differences between the patients with and without TOAs were 1) history of hospitalization for PID: 68% (13/19) vs. 29% (25/85); 2) increased erythrocyte sedimentation rate: 82 vs. 41 mm/h; 3) increased WBC count on admission: 16,200 vs. 14,700/ml; 4) failure to respond to initial antibiotic therapy; and 5) longer hospital stay: 7.8 vs. 4.4 days, respectively. Surgical intervention was required in 3 patients: 2 patients who had TOAs and 1 patient who did not have a TOA by clinical examination or by ultrasound.

*Conclusions:* Despite longer hospital stays and blood tests suggesting more severe disease processes, PID complicated by TOA is usually responsive to intravenous (IV) antibiotic therapy without the need for surgical intervention.


Infectious Diseases in Obstetrics and Gynecology 3:135–139 (1995)
© 1995 Wiley-Liss, Inc.

### Epidemiology and Clinical Outcome of Patients Hospitalized With Pelvic Inflammatory Disease Complicated by Tubo-Ovarian Abscess

**Ying Chan, Winsome Parchment, Joan H. Skurnick, Laura Goldsmith, and Joseph J. Apuzzio**

*Departments of Obstetrics and Gynecology (Y.C., W.P., L.G., J.J.A.) and Public Health and Preventive Medicine (J.H.S.), New Jersey Medical School, Newark, NJ*

Abstract

***Objective:*** **The purpose of this retrospective study was to compare the clinical outcome and characteristics of pelvic inflammatory disease (PID) complicated by tubo-ovarian abscess (TOA) with PID without TOA.**

***Methods:*** **Chart reviews were performed for all PID admissions to the University of Medicine and Dentistry of New Jersey-University Hospital, Newark, NJ, from January 1, 1992, to December 31, 1993.**

***Results:*** **The incidence in this study of TOA based on sonographic evidence of a complex adnexal mass was 18%. The major differences between the patients with and without TOAs were 1) history of hospitalization for PID: 68% (13/19) vs. 29% (25/85); 2) increased erythrocyte sedimentation rate: 82 vs. 41 mm/h; 3) increased WBC count on admission: 16,200 vs. 14,700/ml; 4) failure to respond to initial antibiotic therapy; and 5) longer hospital stay: 7.8 vs. 4.4 days, respectively. Surgical intervention was required in 3 patients: 2 patients who had TOAs and 1 patient who did not have a TOA by clinical examination or by ultrasound.**

***Conclusions:*** **Despite longer hospital stays and blood tests suggesting more severe disease processes, PID complicated by TOA is usually responsive to intravenous (IV) antibiotic therapy without the need for surgical intervention.** © 1995 Wiley-Liss, Inc.

Key words

Surgical intervention, inflammatory mass, intravenous antibiotic therapy

Pelvic inflammatory disease (PID) is a syndrome that may include endometritis, parametritis, salpingitis, oophoritis, pelvic peritonitis, and tubo-ovarian abscess (TOA). About 1 million women per year are treated for acute PID, resulting in an estimated annual cost of more than $4 billion.[1]

TOAs have been reported to occur in as many as 34% of hospitalized patients with PID.[2–4] PID complicated by a TOA carries a poorer prognosis than uncomplicated PID. The presence of a TOA is more likely to require surgical intervention.

The present study reports the epidemiology and clinical outcomes of hospitalized patients with PID.

### MATERIALS AND METHODS

Chart reviews were done for all PID admissions to the University of Medicine and Dentistry of New Jersey-University Hospital, Newark, NJ, from January 1, 1992, to December 31, 1993. All patients met the published criteria for the clinical diagnosis of acute PID.[5] These criteria include lower abdominal tenderness, cervical motion ten-

Address correspondence/reprint requests to Dr. Joseph J. Apuzzio, Department of Obstetrics and Gynecology, New Jersey Medical School, 185 South Orange Avenue, Newark, NJ 07103.

Received April 4, 1995
Accepted September 12, 1995

**TABLE 1. Demographic and historical characteristics of patients hospitalized with PID**

| | PID without TOA (N = 85) | PID with TOA (N = 19) | $P^a$ |
|---|---|---|---|
| Mean age (years) | 25 | 29 | NS |
| Gravity | 3 | 4 | NS |
| Parity | 2 | 2 | NS |
| Prior admission for PID | 25 (29%) | 13 (68%) | 0.0029 |
| History of drug abuse | 20 (24%) | 3 (16%) | NS |
| History of IV drug use | 10 (12%) | 2 (11%) | NS |
| History of alcohol use | 20 (24%) | 1 (5%) | NS |

[a]P value calculated by Student's t-test. NS = not significant.

derness, and bilateral adnexal tenderness, plus 1 or more of the following: temperature ≥38°C, WBC >10,000/ml, purulent material on culdocentesis, evidence of pelvic abscess by ultrasonography or pelvic examination, evidence of gonococcal cervicitis (by Gram's stain showing gram-negative intracellular diplococci), or mucopurulent cervicitis.

A total of 113 PID patients were admitted during the study period. Nine patients were excluded from the study: 4 patients with pyelonephritis, 1 patient with pneumonia, 1 patient with small-bowel obstruction, 1 patient with active pulmonary tuberculosis, 1 patient with an uncertain diagnosis of PID vs. acute appendicitis, and 1 patient with a missing chart.

There were 85 cases of uncomplicated PID and 19 cases of PID complicated by pelvic masses or suspected TOAs. All patients with evidence of a pelvic mass by physical examination or an inadequate pelvic examination underwent sonography. The patients who had sonographic evidence of pelvic masses and those who met the clinical criteria for the diagnosis of PID were categorized as PID complicated by TOA. Human immunodeficiency virus (HIV) testing was offered to all patients, with 25% (26/104) refusing. Endocervical cultures for *Neisseria gonorrhoeae* and *Chlamydia trachomatis* (by Microtrak, Syva Company, San Jose, CA, or Chlamydiazyme, Abbott Laboratories, North Chicago, IL) were performed on all patients. Urine cultures were tested in all patients. A blood culture was obtained if the patient's temperature was >38°C.

All except 7 patients were treated with intravenous (IV) cefoxitin/doxycycline or gentamicin/clindamycin, as recommended by the CDC.[6] Ampicillin was added in a patient with suspected or sonographically proven evidence of a TOA. Imipenem-cilastatin (Primaxin) was used in patients who "failed" ampicillin/gentamicin/clindamycin. Treatment failure was defined as a lack of clinical improvement in symptomatology and persistently elevated WBC counts after 48–72 h of IV antibiotic therapy. Seven patients were treated with ampicillin/sulbactam and doxycycline based on a prior research protocol comparing the efficacy of ampicillin/sulbactam and doxycycline to cefoxitin and doxycycline or gentamicin and clindamycin.

As statistical analysis was performed using Fisher's exact test for discrete variables and Student's t-test for continuous variables. $P < 0.05$ was considered statistically significant.

### RESULTS

The prevalence of TOAs among patients hospitalized for PID at the University Hospital was 18% (19/104). The presenting clinical findings for patients with uncomplicated PID (no inflammatory adnexal mass) and those with TOAs were similar. Table 1 shows that there were no statistically significant differences between these 2 groups in age, gravity, parity, history of drug abuse, or history of alcohol abuse. However, the patients with TOAs had a higher rate of prior hospitalization for PID (68% vs. 29%, respectively; $P = 0.003$).

Table 2 summarizes the laboratory findings on admission. Significantly elevated WBC counts were noted on admission (16,200/ml vs. 14,700/ml, respectively; $P = 0.010$). The erythrocyte sedimentation rates were also significantly elevated in patients with TOAs (82 vs. 41 mm/h, respectively; $P = 0.0002$). The hematocrits were similar in both groups. The results of all endocervical cultures and admitting temperatures were not different between

TABLE 2. Admitting laboratory findings in patients with TOA vs. patients without TOA

| | PID without TOA (N = 85) | PID with TOA (N = 19) | $P$[a] |
|---|---|---|---|
| WBC (mean/ml) | 14,753 | 16,285 | 0.01 |
| Temperature (°C) | 38.39 | 38.44 | NS |
| Hematocrit (%) | 36.8 | 37.2 | NS |
| ESR[b] (mm/h) | 41 | 82 | 0.0002 |
| Endocervical culture | | | |
| GC[c] β-lactamase positive | 6 (7%) | 1 (5%) | NS |
| GC β-lactamase negative | 36 (42%) | 4 (21%) | NS |
| *Chlamydia trachomatis* | 17 (20%) | 2 (10%) | NS |
| Group B streptococcus | 11 (13%) | 2 (11%) | NS |
| *Escherichia coli* | 5 (6%) | 0 | NS |
| *Staphylococcus aureus* | 0 | 1 (5%) | NS |

[a]$P$ value calculated by Student's t-test and Fisher's exact test. NS = not significant.
[b]ESR = erythrocyte sedimentation rate.
[c]GC = *N. gonorrhoeae*.

TABLE 3. Results of HIV testing

| | PID without TOA (N = 85) | PID with TOA (N = 19) | $P$[a] |
|---|---|---|---|
| HIV test accepted | 64 (75%) | 14 (74%) | NS |
| HIV positive | 9 (14%) | 1 (7%) | NS |
| HIV negative | 55 (86%) | 13 (93%) | NS |

[a]$P$ value calculated by Fisher's exact test. NS = not significant.

TABLE 4. Treatment failures and duration of hospital stay

| | PID without TOA (N = 85) | PID with TOA (N = 19) | $P$[a] |
|---|---|---|---|
| Failed initial IV antibiotic therapy | 3 (3.5%) | 8 (42%) | <0.0001 |
| Surgical intervention needed | 1 (1.2%) | 2 (10%) | 0.0851 |
| Mean hospital stay (days) | 4.4 | 7.8 | <0.0001 |

[a]$P$ value calculated by Student's t-test and Fisher's exact test.

the 2 groups. Among the patients with TOAs, 26% (5/19) had *N. gonorrhoeae* (21% being β-lactamase negative and 5% being β-lactamase positive) and 10% (2/19) had *C. trachomatis*. Among the patients without TOAs, *N. gonorrhoeae* was isolated in 49% (42% being β-lactamase negative and 7% being β-lactamase positive) and 20% (17/85) had *C. trachomatis*.

Of all study patients, 75% (78/104) accepted HIV testing. As noted in Table 3, both groups of patients had similar acceptance rates for HIV testing. Of the 75% who had HIV testing, 12.8% (10/78) were found to be HIV positive by enzyme-linked immunosorbent assay (ELISA) and Western blot, 14% (9/64) with uncomplicated PID and 7% (1/14) with TOAs [$P$ = not significant (NS)].

Table 4 is a summary of the treatment failures and durations of hospital stay. Failed initial IV antibiotic therapy was defined as a lack of clinical improvement in symptomatology and persistently elevated WBC counts after 48–72 h of IV cefoxitin/doxycycline or gentamicin/clindamycin therapy. In the group with TOAs, 42% (8/19) failed initial IV antibiotic therapy in comparison with 4% (3/85) of the group without TOAs ($P < 0.0001$). However, most patients in both groups improved clinically when different antibiotics such as ampicillin/gentamicin/clindamycin or imipenem-cilastatin were used. The need for surgical intervention was not significantly different between the 2 groups. Three (2.9%) patients required surgical interventions. One patient with no sonographic evidence of TOA who failed IV antibiotic therapy was HIV negative. She received IV gentamicin/clindamycin for 48 h with no clinical improvement. The antibiotic therapy was then switched to imipenem-cilastatin/doxycycline for another 48 h with no clinical improvement. She underwent an exploratory laparotomy and bilateral salpingectomy. Microabscesses were noted during the surgery, and the histologic findings were consistent with acute suppurative salpingitis. Two patients with TOAs required surgical intervention. Both patients were placed on ampicillin/gentamicin/clindamycin for approximately 48 h, followed by imipenem-cilastatin for >48 h without improvement. Each of these patients underwent a unilateral adnexectomy.

All PID patients were advised to have follow-up visits in the clinic after discharge. Ultrasonography was obtained for every PID patient complicated by a pelvic mass. A surgical resection was advised to each patient with a persistent mass.

The mean hospital stay was longer for patients with TOAs compared with patients without TOAs (7.8 vs. 4.4 days, respectively; $P < 0.0001$).

### DISCUSSION

A TOA is a well-recognized complication of PID. Its etiology is polymicrobial. Three major groups of microorganisms play etiologic roles in PID: *N. gonorrhoeae*, *C. trachomatis*, and a wide variety of anaerobic and aerobic bacteria. Although our study showed no differences in bacteriology between complicated and uncomplicated PID, anaerobic cultures were not done in the majority of the patients. The exact mechanism of TOA formation is difficult to establish.[7] Studies have shown that pathogens can ascend to the fallopian tube, attach themselves to mucosal epithelial cells, penetrate the epithelial cells through phagocytosis, and cause destruction of the epithelial cells, resulting in purulent exudate production.[8,9] The abscess may remain localized, involving only the tube and ovary, or it may involve the uterus, bowel, bladder, or opposite adnexa.

PID complicated by a TOA carries a significantly higher morbidity and mortality than uncomplicated PID. In this study, only 68% (13/19) of the patients with TOAs had histories of PID, which may be explained by the possibility of a previous subclinical infection or by the presence of certain pathogens that are more likely to cause abscess formation.[3]

There are many etiologies for a pelvic mass, including follicular cyst, ovarian neoplasm, uterine leiomyoma, hydrosalpinx, and TOA. A large number of retrospective studies have evaluated the accuracy of ultrasonography in the diagnosis of a pelvic abscess. Landers and Sweet[10] reported on a series of 31 patients with surgically confirmed TOAs who were evaluated with ultrasonography. Of the 31 surgically confirmed cases, 29 were reported as complex adnexal masses or cystic-type masses with multiple internal echoes by ultrasound. Only 2 patients had surgically proven TOAs in our group of patients with presumed PID complicated by TOAs. All of the other patients met the clinical criteria for PID as well as pelvic masses consistent with TOAs by ultrasonography.

HIV testing is strongly advisable in all patients with PID. The incidence of HIV seropositivity was 12.8% (10/78) among our patients hospitalized with PID. This rate is comparable to the 13.6% rate reported by investigators of Kings County Hospital Center in Brooklyn, NY.[11] However, investigators from San Francisco General Hospital reported the HIV seropositivity rate to be only 6.7% in patients with PID.[12] Hoegsberg et al.[12] reported no statistically significant difference in surgical intervention between HIV-seropositive and HIV-seronegative patients. Our study supports this finding. Furthermore, the incidence of TOA was similar in these 2 groups of patients: 14% (9/64) of the patients with uncomplicated PID and 7% (1/14) with complicated PID were HIV positive ($P$ = NS). However, since only 10 HIV-positive patients were identified in the study and the power of the study was limited, a difference in clinical outcome may be found when more patients are studied.

There is general agreement that a ruptured TOA requires surgical intervention. In the past, prompt surgical intervention with complete removal of the uterus and adnexa was performed.[13–15] Nevertheless, because of intraoperative complications such as bowel injury,[14] loss of reproductive and ovarian functions, a total abdominal hysterectomy and bilateral salpingo-oophorectomy may not be an optimal treatment. In our series of 104 patients, most patients with TOAs improved with IV antibiotics. No statistically significant difference was noted in surgical intervention between these 2 groups. Therefore, we believe that conservative medical therapy plays an important role in the treatment of PID complicated by TOA. In a patient with a unilateral TOA unresponsive to IV antibiotics, a unilateral adnexectomy is the procedure of choice if she desires future fertility.

In conclusion, the major differences between the PID patients with and without TOAs were 1) history of hospitalization for PID, 2) increased erythrocyte sedimentation rate, 3) increased WBC count on admission, 4) failure to respond to initial antibiotic therapy, and 5) longer hospital stay. No differences were noted in age, parity, drug or alcohol use, results of endocervical cultures, HIV seropositivity rate, or need for surgical intervention.

These results suggest that a patient with PID complicated by a TOA suspected by ultrasound is usually responsive to IV antibiotics without the need for surgical intervention.

### REFERENCES

1. Washington AE, Katz P: Cost of and payment source for pelvic inflammatory disease: Trends and projections, 1983 through 2000. JAMA 266:2565–2569, 1991.
2. Hager WD: Follow-up of patients with tuboovarian abscesses in association with salpingitis. Obstet Gynecol 61: 680, 1983.
3. Benigno BB: Medical and surgical management of the pelvic abscess. Clin Obstet Gynecol 24:1187, 1981.
4. Franklin EW, Hevron JE, Thompson JD: Management of the pelvic abscess. Clin Obstet Gynecol 16:66, 1973.
5. Landers DV, Sweet RL: Current trends in the diagnosis and treatment of tuboovarian abscess. Am J Obstet Gynecol 151:1098–1110, 1985.
6. Centers for Disease Control and Prevention: 1993 sexually transmitted diseases treatment guidelines. MMWR 42:75–83, 1993.
7. Hager DW, Eschenbach DA, Spencer M, Sweet RL: Criteria for diagnosis and grading of salpingitis. Obstet Gynecol 61:113–114, 1983.
8. Ward ME, Watt PJ, Robertson JN: The human fallopian tube: A laboratory model for gonococcal infection. J Infect Dis 129:650, 1974.
9. Carney FE, Taylor-Robinson D: Growth and effect of *Neisseria gonorrhoeae* in organ cultures. Br J Vener Dis 49:435, 1973.
10. Landers DV, Sweet RL: Tuboovarian abscess: Contemporary approach to management. Rev Infect Dis 5:876, 1983.
11. Gregg CR, Melly MA, McGee ZA: Gonococcal lipopolysaccharide: A toxin for human fallopian tube mucosa. Am J Obstet Gynecol 138:981–984, 1980.
12. Hoegsberg B, Abulafia O, Sedlis A, et al.: Sexually transmitted diseases and human immunodeficiency virus infection among women with pelvic inflammatory disease. Am J Obstet Gynecol 163:1135–1139, 1990.
13. Safrin S, Dattel BJ, Hauer L, Sweet RL: Seroprevalence and epidemiologic correlates of human immunodeficiency virus infection in women with acute pelvic inflammatory disease. Obstet Gynecol 75:667–670, 1990.
14. Nebel WA, Lucas WE: Management of tuboovarian abscess. Obstet Gynecol 32:381, 1968.
15. Kaplan AL, Jacobs WM, Ehresman JB: Aggressive management of pelvic abscess. Am J Obstet Gynecol 98:482, 1967.
